# Photocatalytic Hydrogen Production by the Sensitization of Sn(IV)-Porphyrin Embedded in a Nafion Matrix Coated on TiO_2_

**DOI:** 10.3390/molecules27123770

**Published:** 2022-06-11

**Authors:** Sung-Hyun Kim, Hee-Joon Kim

**Affiliations:** Department of Chemistry and Bioscience, Kumoh National Institute of Technology, Gumi 39177, Korea; kashik@naver.com

**Keywords:** hydrogen production, photosensitization, Sn(IV)-porphyrin, Nafion, photocatalyst

## Abstract

Efficient utilization of visible light for photocatalytic hydrogen production is one of the most important issues to address. This report describes a facile approach to immobilize visible-light sensitizers on TiO_2_ surfaces. To effectively utilize the sensitization of Sn(IV) porphyrin species for photocatalytic hydrogen production, perfluorosulfonate polymer (Nafion) matrix coated-TiO_2_ was fabricated. Nafion coated-TiO_2_ readily adsorbed *trans*-diaqua[*meso*-tetrakis(4-pyridinium)porphyrinato]tin(IV) cation [(TPy*^H^*P)Sn(OH_2_)_2_]^6+^ via an ion-exchange process. The uptake of [(TPy*^H^*P)Sn(OH_2_)_2_]^6+^ in an aqueous solution completed within 30 min, as determined by UV-vis spectroscopy. The existence of Sn(IV) porphyrin species embedded in the Nafion matrix coated on TiO_2_ was confirmed by zeta potential measurements, UV-vis absorption spectroscopy, TEM combined with energy dispersive X-ray spectroscopy, and thermogravimetric analysis. Sn(IV)-porphyrin cationic species embedded in the Nafion matrix were successfully used as visible-light sensitizer for photochemical hydrogen generation. This photocatalytic system performed 45% better than the uncoated TiO_2_ system. In addition, the performance at pH 7 was superior to that at pH 3 or 9. This work revealed that Nafion matrix coated-TiO_2_ can efficiently produce hydrogen with a consistent performance by utilizing a freshly supplied cationic Sn(IV)-porphyrin sensitizer in a neutral solution.

## 1. Introduction

Photochemical generation of hydrogen has been intensively studied as a means of converting solar energy into chemical energy [1,2,3,4,5,6]. Solar energy is predominantly in the visible region, therefore, efficient utilization of visible light is one of the most important issues to address. During natural photosynthesis, the absorption of visible light by chlorophyll sensitizers initiates the light-harvesting process, followed by charge separation and transfer, which proceeds through redox reactions. Porphyrins and metalloporphyrins have been extensively explored in the context of light harvesting and photoinduced electron/energy transfer processes [7,8,9], because of their similarity to chlorophyll sensitizers in natural photosynthesis. They have also been extensively investigated for their photochemical properties in environmental photocatalysis [10], hydrogen production [11,12], and solar cell [13] applications.

Among the metalloporphyrins, Sn(IV)-porphyrin is particularly noteworthy as a photosensitizer or photocatalyst for the development of various photocatalytic systems. Sn(IV)-porphyrin has an intrinsically strong oxidation ability owing to the high charge of the Sn(IV) center; consequently, the excited state of SnP has a high affinity for electrons that initiate photooxidative reactions. The excited Sn(IV)-porphyrin exhibits a high photochemical activity for the oxidative degradation of organic pollutants under visible light [14,15,16,17,18,19]. Sn(IV)-porphyrin complex-based nanoparticles have been also used in photochemical hydrogen production [20]. A water-soluble Sn(IV)-porphyrin complex, *trans*-diaqua[*meso*-tetrakis(4-pyridinium)porphyrinato]tin(IV) hexanitrate [(TPy*^H^*P)Sn(OH_2_)_2_](NO_3_)_6_, has been investigated as a visible light sensitizer of platinized TiO_2_ nanoparticles for the production of hydrogen [21]. Although the Sn(IV)-porphyrin sensitizer was not bound to TiO_2_, hydrogen was successfully generated under visible light over a wide pH range (pH 3–11). Efficient visible light sensitization generally requires strong chemical bonding between the semiconductor oxide (TiO_2_) and sensitizer molecule, which results in significant electronic coupling between the semiconductor conduction band and the sensitizer’s excited orbital. Therefore, molecular sensitizers with anchors such as carboxylate, phosphonate, and catechol groups are fixed on the surface of the semiconductor oxide [22,23,24]. In contrast, this study revealed that the sufficiently long lifetime of photogenerated π-radical anions of Sn(IV)-porphyrin (SnP^•−^) enables the diffusion of SnP^•−^ to the TiO_2_ surface in the bulk solution. The disadvantage of the typical molecular dye-sensitized TiO_2_ system, in which the chemical modification of the sensitizer for anchoring is essential and the hydrogen production is limited to acidic conditions, was addressed in this system. However, the efficiency of the electron transfer between the porphyrin sensitizer and redox mediator remains to be improved.

An alternative method of immobilizing sensitizing molecules on the surface of TiO_2_ has been achieved using a polymer matrix [25,26]. Nafion, an anionic perfluorinated polymer, has hydrophilic pores (~4 nm) surrounded by sulfonate anion groups (−SO_3_^−^) capable of exchanging cationic species. Additionally, it is chemically and photochemically inert. [(TPy*^H^*P)Sn(OH_2_)_2_]^6+^, which does not bind to the TiO_2_ surface, can be embedded into the Nafion-coated TiO_2_ surface through ion exchange, which facilitates more efficient production of hydrogen under visible light.

**Scheme 1:**
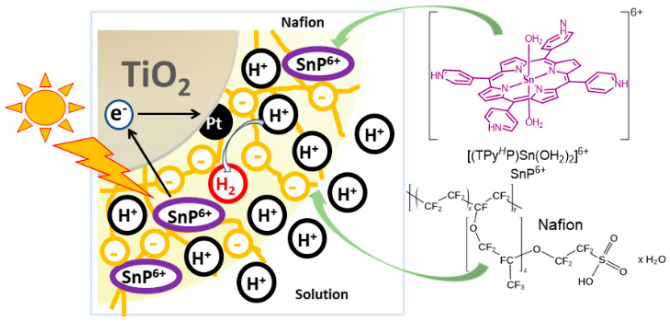
Illustration for the photochemical hydrogen generation by sensitization of Sn(IV)-porphyrin embedded in Nafion matrix coated on TiO_2_.

Unlike Ru-based sensitizers, Sn(IV)-porphyrins can be developed and used as practical sensitizers for solar energy conversion because they are inexpensive, have low toxicity, and are rich in certain elements. To improve the efficiency of visible-light-sensitive hydrogen generation using porphyrin sensitizers, we investigated a photocatalytic hydrogen generation system that incorporated Sn(IV)-porphyrin cations into a perfluorosulfonate polymer (Nafion) matrix coated on platinized TiO_2_ nanoparticles (Scheme 1).

## 2. Results and Discussion

### 2.1. Fabrication of Photocatalyst

**Scheme 2:**
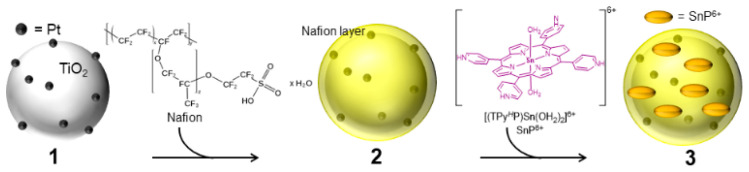
Fabrication of photocatalysts used in this study.

**Figure 1:**
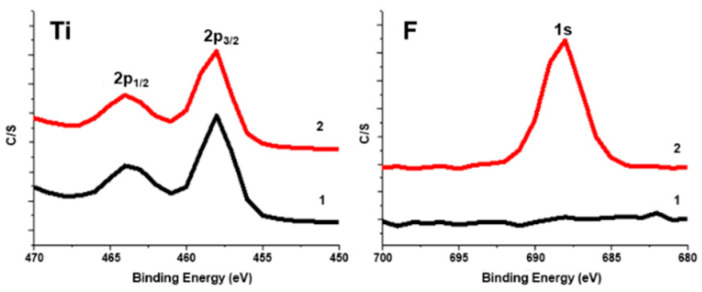
XPS spectra of platinized TiO_2_ (**1**) and Nafion-coated Pt-TiO_2_ (**2**) in the Ti 2p (left) and F 1s band regions.

Scheme 2 illustrates the fabrication of photocatalysts used in this study. Platinized TiO_2_ nanoparticles (**1**) were prepared by the chemical reduction of H_2_PtCl_6_ with NaBH_4_. The TEM image, EDS spectrum, and elemental mapping images obtained by STEM showed the presence of Pt particles on the TiO_2_ surface (Figure S1). The Nafion polymer was easily coated onto the Pt-TiO_2_ surface by the drop-casting method using a commercial Nafion solution. Nafion-coated Pt-TiO_2_ (**2**) was characterized by TEM, EDS, X-ray photoelectron spectroscopy (XPS), and FT-IR techniques. The EDS spectrum indicated the presence of F and S elements in the Nafion layer of **2** (Figure S2). The XPS spectra as shown in Figure 1 clearly confirmed the presence of F (1 s binding energy of 687 eV) in **2**, but not in **1**. The Ti 2p binding energies (464 and 458 eV) of both **2** and **1** were identical to those of pure TiO_2_. In the IR spectra comparing **2** and **1** (Figure S3), the C–F vibration band was observed at 1239 cm^−1^ only from **2** further supporting the presence of the Nafion layer. Therefore, all the characterization data prove the successful fabrication of **2**.

### 2.2. Embedding of Sn(IV)-Porphyrin Cations into Nafion-Coated Photocatalyst

**Figure 2:**
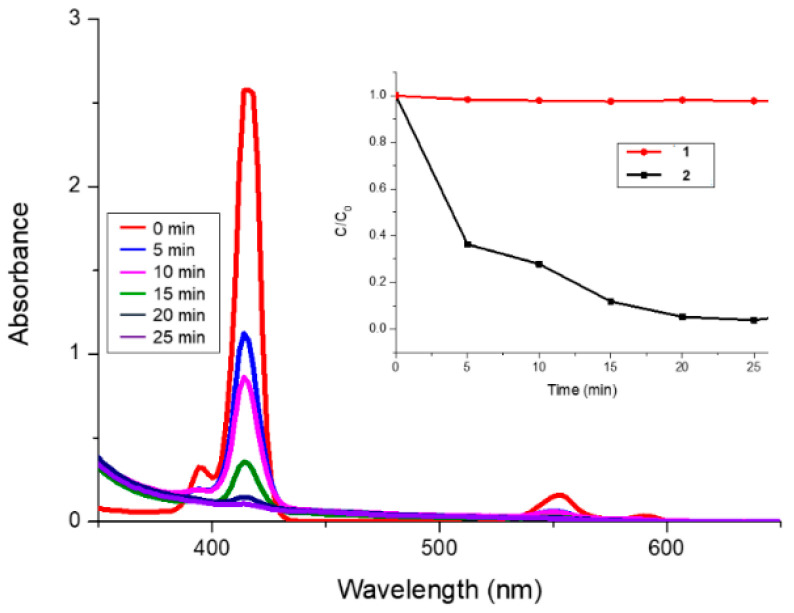
UV-vis absorption spectra of SnP^6+^ remaining after the equilibrated adsorption on Nafion-coated Pt-TiO_2_ (**2**). The inset depicts the comparison of absorption changes in Soret bands of SnP^6+^ according to adsorption progress with time on **1** and **2**.

The adsorption of ionic surfactant polymers, such as Nafion, on the TiO_2_ surface can modify the surface charge drastically. The zeta potential of suspended **2** at pH 7, −28.5 mV, was measured to be more negative than that of **1**, −8.8 mV, indicating that the anionic character due to the coated Nafion layer was significantly manifested on the surface of particles of **1**. **2** was expected to readily adsorb certain cationic species through an ion-exchange process in the Nafion matrix. The uptake of [(TPy*^H^*P)Sn(OH_2_)_2_]^6+^ (SnP^6+^, water-soluble and highly charged Sn(IV)-porphyrin cation) by **2** suspended in an aqueous solution was monitored using UV-vis spectroscopy. Figure 2 shows that the absorption in the Soret band of SnP^6+^ decreased gradually, and the uptake was completed within 30 min. In contrast, **1** (uncoated Nafion polymer) did not adsorb SnP^6+^ at all, evident by the unchanged absorption spectra. The inset in Figure 2 also shows the uptake of SnP^6+^ quantitatively over time.

**Figure 3:**
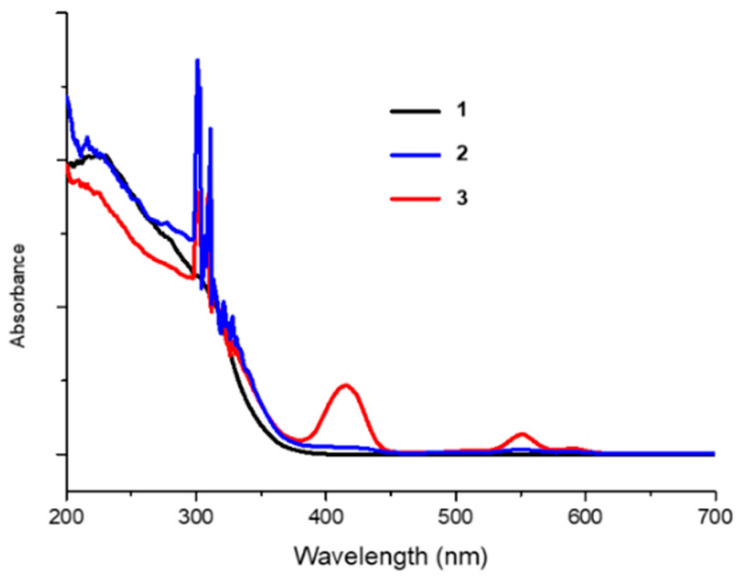
UV-vis absorption spectra of platinized TiO_2_ (**1**), Nafion-coated Pt-TiO_2_ (**2**), and SnP^6+^-embedded Nafion/Pt-TiO_2_ (**3**) measured in solid state.

Isolated **2** containing SnP^6+^ (**3**) was further characterized. In the zeta potential measurement, **3** showed +20.9 mV at pH 7, which strongly implies SnP^6+^ was sufficiently incorporated into the Nafion matrix of **2**. In contrast, that of **1** was measured to be −14.5 mV when SnP^6+^ was present in aqueous solution, which indicates that **1** itself does not adsorb SnP^6+^ in aqueous solution. Figure 3 shows the UV-vis absorption spectra for each nanoparticle measured in the solid state. When compared to **1** and **2**, the spectrum of **3** exhibited strong absorption bands from SnP^6+^ at 414, 511, and 550 nm in the visible light region. The TEM image and the EDS spectrum for **3** further proves the existence of key elements such as Sn, S, F, Pt, and Ti constituting **3** (Figure S4).

**Figure 4:**
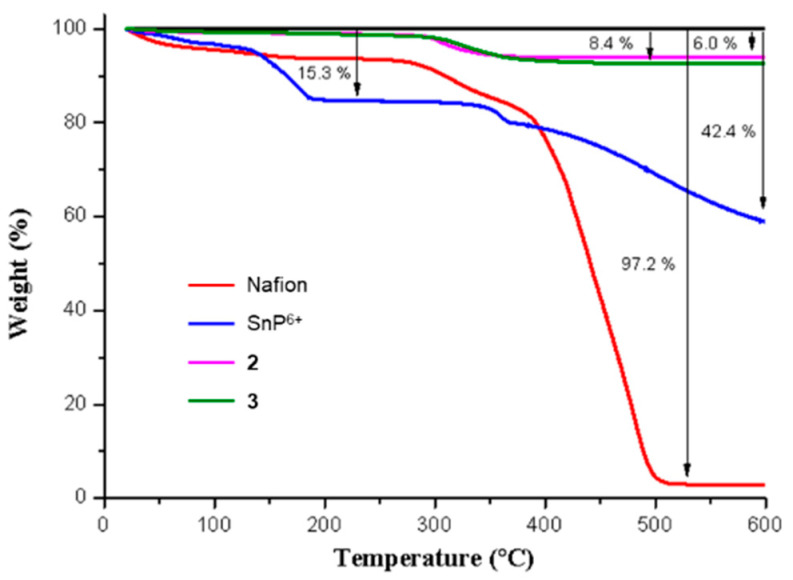
TGA thermogram of SnP^6+^-embedded Nafion/Pt-TiO_2_ (**3**).

Finally, TGA measurements were taken to determine the content of SnP^6+^ in **3** (Figure 4). In the TGA diagram of [(TPy*^H^*P)Sn(OH_2_)_2_](NO_3_)_6_, the removal of solvent molecules such as water occurred up to ~150 °C, decomposition of nitrate anions occurred at 200–350 °C, followed by the degradation of the Sn(IV)-porphyrin. In addition, the TGA plot of the Nafion sample showed that Nafion gradually lost water molecules and began to decompose rapidly at 300 °C, eventually losing 97 wt.% at 500 °C. **2** and **3** exhibited similar behavior in TGA, where the final plateau was achieved above 500 °C for both samples. Based on the difference of weight loss between **2** and **3**, the content of SnP^6+^ was estimated to be about 2.4 wt.% in bulk **3**.

### 2.3. Photocatalytic Hydrogen Generation

**Figure 5:**
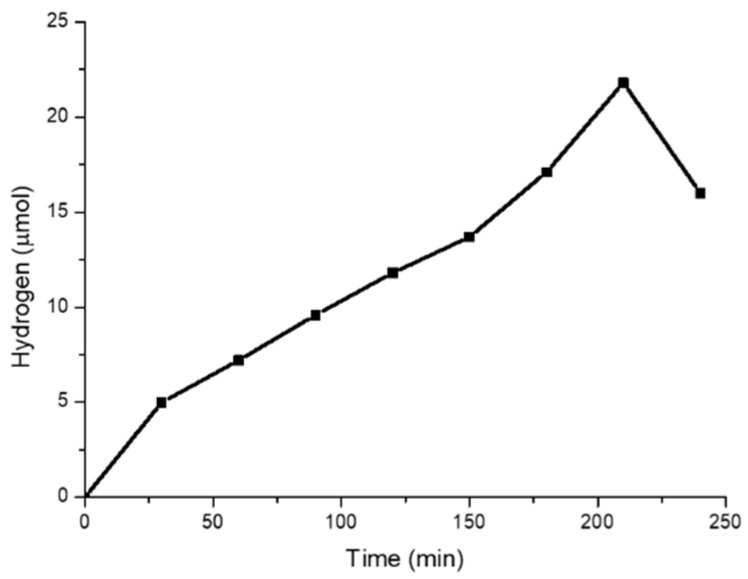
Time profile of H_2_ production using the photocatalyst of SnP^6+^-embedded Nafion/Pt-TiO_2_ (**3**) in the absence of SnP^6+^ in the solution under visible light irradiation.

Photocatalytic hydrogen generation was first investigated under visible light irradiation in an aqueous suspension containing **3** (2.4 wt.% of SnP^6+^, 1.5 g/L) as the photocatalyst and EDTA (1 mM) as the electron donor, but without an additional SnP^6+^ sensitizer. As shown in Figure 5, the amount of hydrogen generated continuously increased to 22 μmol after 4 h. It was clearly demonstrated that the SnP^6+^ species embedded in the Nafion matrix functioned successfully as a visible light sensitizer for photochemical hydrogen generation. The decrease in hydrogen production after 4 h may be due to the irreversible conversion of Sn(IV)-porphyrin to Sn(IV)-chlorin, a reduced form of the pyrrole ring [21].

**Figure 6:**
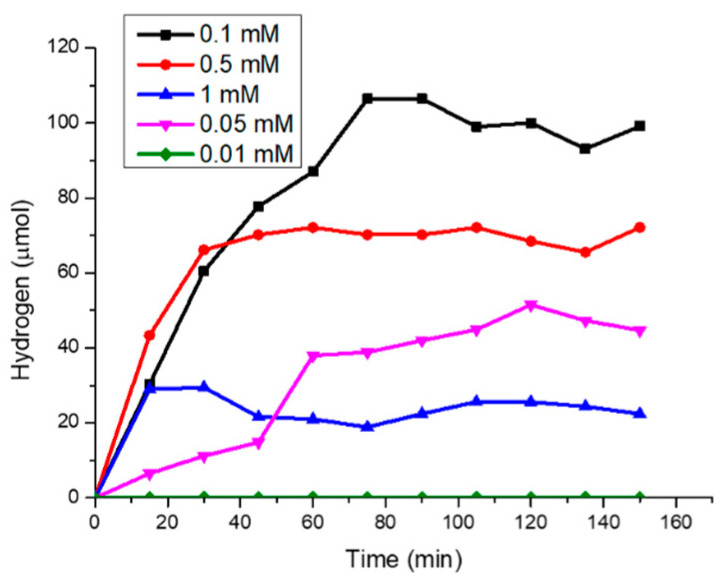
Comparison of visible light-irradiated hydrogen production of Nafion-coated Pt-TiO_2_ (**2**) by varying the concentration of SnP^6+^.

The effect of SnP^6+^ concentration on hydrogen generation in a suspension of **1** was examined, as displayed in Figure 6. While the amount of hydrogen generated was negligible at 0.01 mM concentration, increased remarkably as the concentration of the sensitizer increased to 0.1 mM. This means that the sensitizer in the solution must exceed a certain critical concentration to enter the Nafion matrix. On the other hand, at higher concentrations (0.5 and 1.0 mM), the hydrogen production increased sharply, but was prematurely saturated at approximately 30–45 min irradiation, and the amount produced was substantially less than that 0.1 mM concentration after further irradiation. This revealed that a large excess of the SnP^6+^ sensitizer can initially enhance the rate of incorporation into the Nafion matrix, but the surplus in solution has little effect on hydrogen production. Photocatalyst **2** itself does not contain SnP^6+^ sensitizer, so it is crucial to uptake SnP^6+^ sensitizer from solution at an initial stage. The uptake rate and efficiency probably depend on the concentration and mass transfer of SnP^6+^. Consequently, the optimal concentration of the SnP^6+^ sensitizer could efficiently incorporate the sensitizer into the Nafion matrix to subsequently promote photosensitized hydrogen generation.

**Figure 7:**
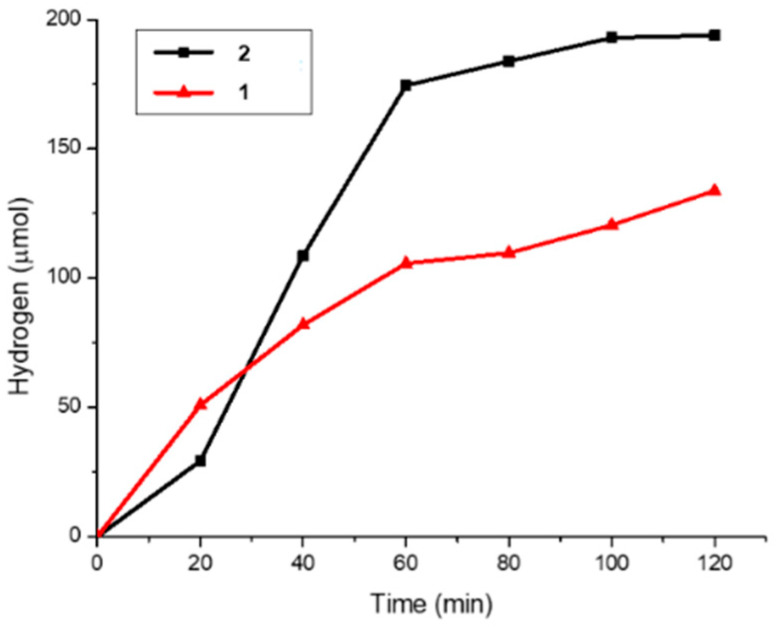
Comparison of visible light-irradiated hydrogen production in the presence of 0.1 mM SnP^6+^ of platinized TiO_2_ (**1**) and Nafion-coated Pt-TiO_2_ (**2**).

The adsorption of SnP^6+^ on TiO_2_ is not required for photocatalytic H_2_ production, hence, we further compared the performance of **2** with that of **1** in the presence of 0.1 mM SnP^6+^. As shown in Figure 7, 193 and 133 μmol of hydrogen were generated by the photocatalysts **2** and **1**, respectively, after 2 h of irradiation. **2** exhibited a 45% better performance than uncoated TiO_2_. SnP^6+^ sensitizers embedded into the Nafion matrix coated on the surface of TiO_2_ facilitate the electron transfer process between the sensitizer and redox mediator (TiO_2_) when compared to free SnP^6+^ sensitizers in solution. The higher local concentration of H^+^ in the Nafion matrix also contributed significantly. The H^+^ population on the Nafion polymer-coated TiO_2_ surface increased considerably owing to the presence of the sulfonate groups in the Nafion polymer. It is well known that the pH of Nafion is much lower than that of the aqueous bulk phase. The protons trapped in the Nafion matrix of **2** could then be readily photochemically reduced to form hydrogen. Accordingly, the performance of **2** for the photocatalytic H_2_ generation was enhanced compared to that of **1**.

**Figure 8:**
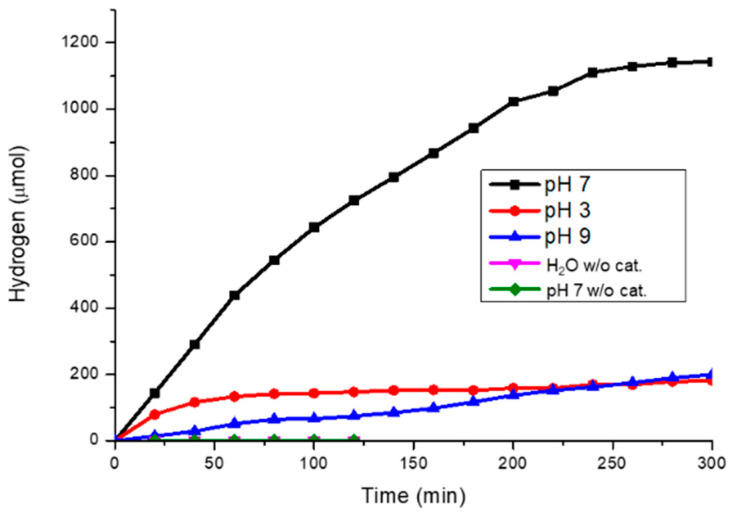
Comparison of visible light-irradiated hydrogen production of Nafion-coated Pt-TiO_2_ (**2**) at different pH values in the presence of 0.1 mM SnP^6+^.

In a previous report [21], the unbound SnP^6+^-sensitized TiO_2_ system was found to successfully generate hydrogen under visible-light irradiation over a wide pH range (pH 3–11). Here, we investigated photocatalytic hydrogen generation with SnP^6+^-sensitized **2** at different pH values to evaluate the effect of pH on the performance and stability of the photocatalyst. Figure 8 shows the performance of H_2_ generation sensitized by SnP^6+^ in **2** at three different pH values (3, 7, and 9).

The performance at pH 7 was superior to that at pH 3 or 9, where a similar performance was observed. At pH 3, hydrogen production gradually increased in the initial stage but almost ceased after 1 h. The photocatalytic production of hydrogen through the sensitization of Sn(IV)-porphyrin species is affected by the action of the corresponding π-radical anion species. Sn(IV)-chlorin, a reduced form of the pyrrole ring, is irreversibly formed by a bimolecular reaction between π-radical anions. The favorable formation of Sn(IV)-chlorin at an acidic pH inhibits the electron transfer process from the π-radical anions to the TiO_2_ or platinum catalyst, thereby reducing hydrogen production performance. It was also observed that the Nafion matrix coated on the TiO_2_ peeled off at pH 9 and above. This exfoliation can explain why hydrogen production under basic conditions did not increase as much as that at neutral pH over time. Therefore, it can be concluded that Nafion matrix coated-TiO_2_ can efficiently produce hydrogen with a consistent performance by utilizing a freshly supplied cationic Sn(IV)-porphyrin sensitizer in a neutral solution.

## 3. Materials and Methods

*Trans*-diaqua [5,10,15,20-tetrakis(4-pyridinium)porphyrinato]tin(IV) hexanitrate, [(TPyHP)Sn(OH_2_)_2_](NO_3_)_6_, was prepared using a reported procedure [27]. TiO_2_ nanoparticles (Degussa P25) were used as received. Nafion was purchased from Aldrich as a 5 wt. % solution in a mixture of alcohol and water. Chloroplatinic acid (H_2_PtCl_6_·6H_2_O) (Aldrich, St. Louis, MO, USA), methanol (Aldrich), and ethylenediaminetetraacetic acid (EDTA, Aldrich) were used as received. HClO_4_ and NaOH were used to adjust the pH of aqueous suspensions. Ultrapure deionized water (18 MΩ⋅cm) and was prepared using the Barnstead purification system. Transmission electron microscopy (TEM), TEM-energy dispersive X-ray spectroscopy (TEM-EDS), and scanning transmission electron microscopy (S-TEM) images were obtained using a JEOL/JEM 2100 instrument. The zeta potentials of the catalyst particles in the aqueous suspension were measured using an electrophoretic light-scattering spectrophotometer (ELSZ-2, Otsuka, Osaka, Japan). The surface atomic composition was determined using X-ray photoelectron spectroscopy (XPS, ULVAC-PHI/Quantera). UV-visible spectra were recorded using a UV-vis spectrophotometer (UV-3600, Shimadzu, Tokyo, Japan). FT-IR spectra were recorded in the range of 4000–400 cm^−1^ on a Bruker Vertex 80v. Thermogravimetric analyses (TGA) were carried out on a TA Instruments/Auto-TGA Q502 instrument heated from room temperature to 600 °C at a ramp rate of 5 °C/min under nitrogen.

### 3.1. Preparation of Photocatalyst

#### 3.1.1. Platinized TiO_2_ (**1**)

A 2.0 g sample of TiO_2_ nanoparticles were immersed in water with 100 mL of H_2_PtCl_6_·H_2_O (0.1 M) while being continuously stirred for 2 h. Then, 50 mL of NaBH_4_ (1.0 M NaBH_4_ in methanol) was added quickly and stirred continuously for 2 h. The photocatalyst color changed from white to black with increasing Pt loading. The powder was washed repeatedly with distilled water. The suspension was centrifuged and decanted. The residue was then dried overnight at 90 °C, and this yielded (2.2 g of **1**).

#### 3.1.2. Nafion-Coated Pt-TiO_2_ (**2**)

An aliquot of Nafion solution (2 mL) in H_2_O/MeOH was added to **1** (1.0 g), and the mixture was mixed thoroughly. The suspension was centrifuged and decanted. The residue was washed with H_2_O/MeOH, and dried overnight at 90 °C, and this yielded (1.2 g of **2**).

#### 3.1.3. Sn(IV)-porphyrin cations-embedded Nafion/Pt-TiO_2_ (**3**)

An aliquot of 0.1 mL of a 1.0 mM [(TPy*^H^*P)Sn(OH_2_)_2_](NO_3_)_6_ solution in H_2_O was added to **2** (0.1 g), and the reaction mixture was vigorously stirred for 1 h. The suspension was centrifuged and decanted. The residue was dried overnight at 90 °C, and this yielded (0.1 g of **3**).

### 3.2. Photocatalytic Hydrogen Generation

**2** (7.5 mg, 1.5 g/L) was suspended in an aqueous solution of SnP^6+^ (0.1 mM) and EDTA (1 mM) in a glass reactor (20 mL, Wheaton, Stoke-on-Trent, UK). The mixture was vigorously stirred for 1 h to immobilize SnP^6+^ on the Nafion layer. The suspension was purged with N_2_ for 1 h before illumination. A 150 W xenon arc lamp was used as the light source (LS 150, ABET-technologies, Milford, CT, USA). Light was passed through a 10-cm IR cut-off filter (λ > 900 nm, Edmund Optics, Barrington, IL, USA) and a UV cut-off filter (λ < 400 nm, Edmund Optics), and the headspace gas (15 mL) of the reactor was intermittently sampled and analyzed for hydrogen using a gas chromatograph (GC-2014, Shimadzu, Tokyo, Japan).

## 4. Conclusions

Perfluorosulfonate polymer (Nafion) matrix coated-TiO_2_ was fabricated to effectively sensitize Sn(IV)-porphyrin species for photocatalytic hydrogen production. Nafion coated-TiO_2_ readily adsorbed Sn(IV)-porphyrin cation species via an ion-exchange process. The presence of the Sn(IV)-porphyrin species embedded in the Nafion matrix coated on TiO_2_ was confirmed using various instrumental techniques. Our investigation revealed that the Sn(IV)-porphyrin cationic species embedded in the Nafion matrix successfully functioned as a visible-light sensitizer for photocatalytic hydrogen generation. This photocatalytic system performed 45% better than the uncoated TiO_2_ system. In addition, the performance at pH 7 is much better than that at pH 3 or 9. In conclusion, Nafion matrix coated-TiO_2_ can efficiently produce hydrogen through the favorable uptake of cationic Sn(IV)-porphyrin sensitizer in a neutral solution. Our work makes an important contribution in the development of nanostructured photocatalysts that are more efficient and practical than Ru-based sensitization for visible-light-sensitized hydrogen production.

## Data Availability

Data are available in the article and Supplementary Materials.

## Supplementary Materials

The following supporting information can be downloaded at: https://www.mdpi.com/article/10.3390/molecules27123770/s1. Figure S1: Micrographs for platinized TiO_2_ (**1**) showing (a) TEM image, (b) EDS spectrum, and (c) elemental mapping images by STEM for platinum (left, red), titanium (center, green) and oxygen (right, blue). Figure S2: Micrographs for Nafion-coated Pt-TiO_2_ (**2**) showing (a) TEM image, and (b) EDS spectrum. Figure S3: FT-IR spectra of platinized TiO_2_ (**1**) and Nafion-coated Pt-TiO_2_ (**2**). Figure S4: Micrographs for SnP^6+^-embedded Nafion/Pt-TiO_2_ (**3**) showing (a) TEM image, and (b) EDS spectrum.

## Sample Availability

Samples of the compounds are not available from the authors.
