# Supplementary material for: Photocatalytic Hydrogen Production by the Sensitization of Sn(IV)-Porphyrin Embedded in a Nafion Matrix Coated on TiO2
Source: Molecules. 2022 Jun 11;27(12):3770. doi: 10.3390/molecules27123770 (PMC9227469; doi:10.3390/molecules27123770)
Supplement: Supplementary file 1 [file molecules-27-03770-s001.zip › molecules-1765783-supplementary.pdf]

molecules

Supplementary Materials

Photocatalytic Hydrogen Production by the Sensitization of Sn(IV)-Porphyrin Embedded in a Nafion Matrix Coated on TiO_2

Sung Hyun Kim and Hee-Joon Kim *

Department of Chemistry and Bioscience, Kumoh National Institute of Technology, Gumi 39177, Korea; kashik@naver.com
* Correspondence: hjk@kumoh.ac.kr; Tel.: +82-54-478-7822

List of contents:

Figure S1. Micrographs for platinized TiO_2 (**1**) showing (a) TEM image, (b) EDS spectrum, and (c) elemental mapping images by STEM for platinum (left, red), titanium (center, green) and oxygen (right, blue).

Figure S2. Micrographs for Nafion-coated Pt-TiO_2 (**2**) showing (a) TEM image, and (b) EDS spectrum.

Figure S3. FT-IR spectra of platinized TiO_2 (**1**) and Nafion-coated Pt-TiO_2 (**2**).

Figure S4. Micrographs for SnP^{6+}-embedded Nafion/Pt-TiO_2 (**3**) showing (a) TEM image, and (b) EDS spectrum.

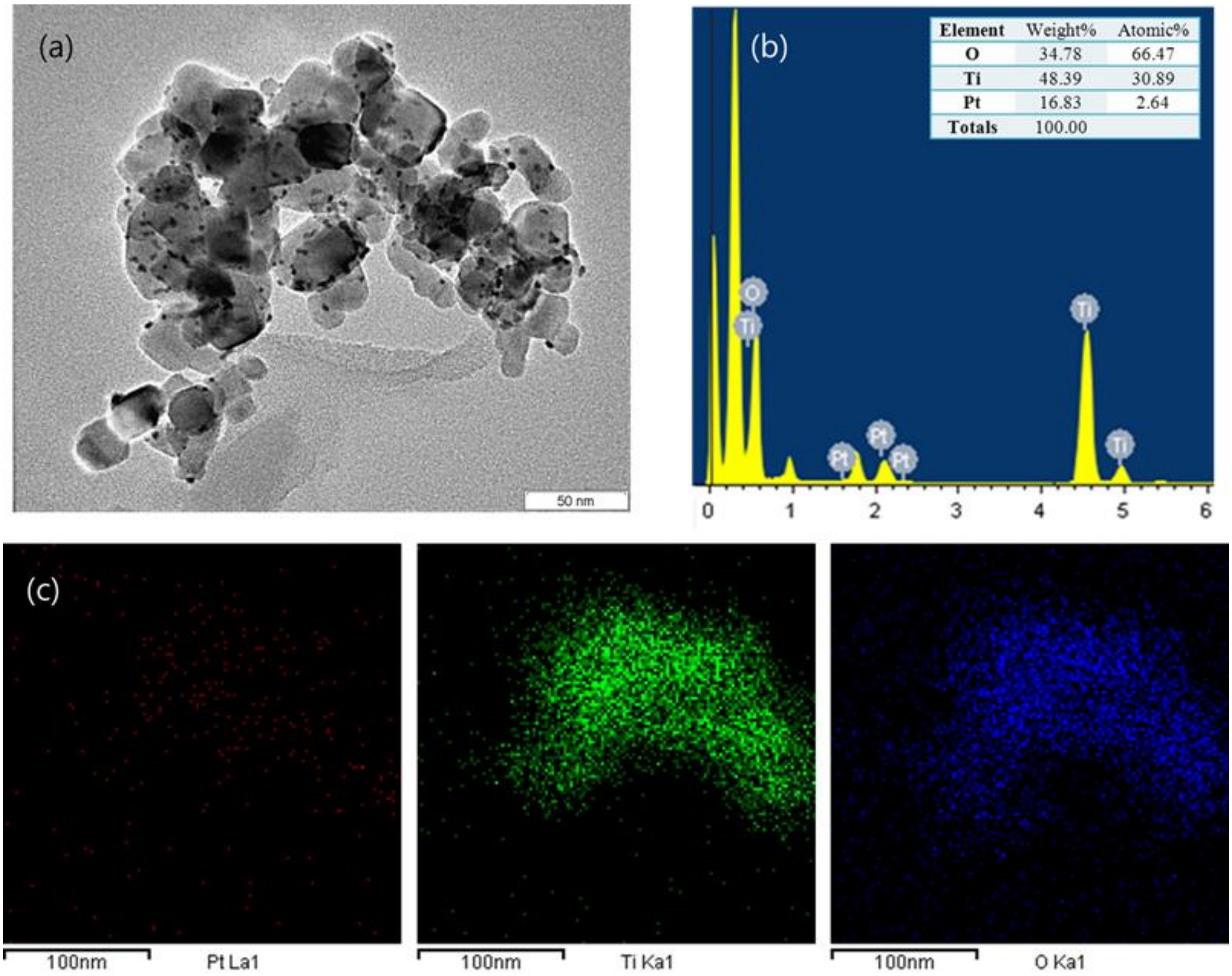

Figure S1. Micrographs for platinized TiO_2 (**1**) showing (a) TEM image, (b) EDS spectrum, and (c) elemental mapping images by STEM for platinum (left, red), titanium (center, green) and oxygen (right, blue).

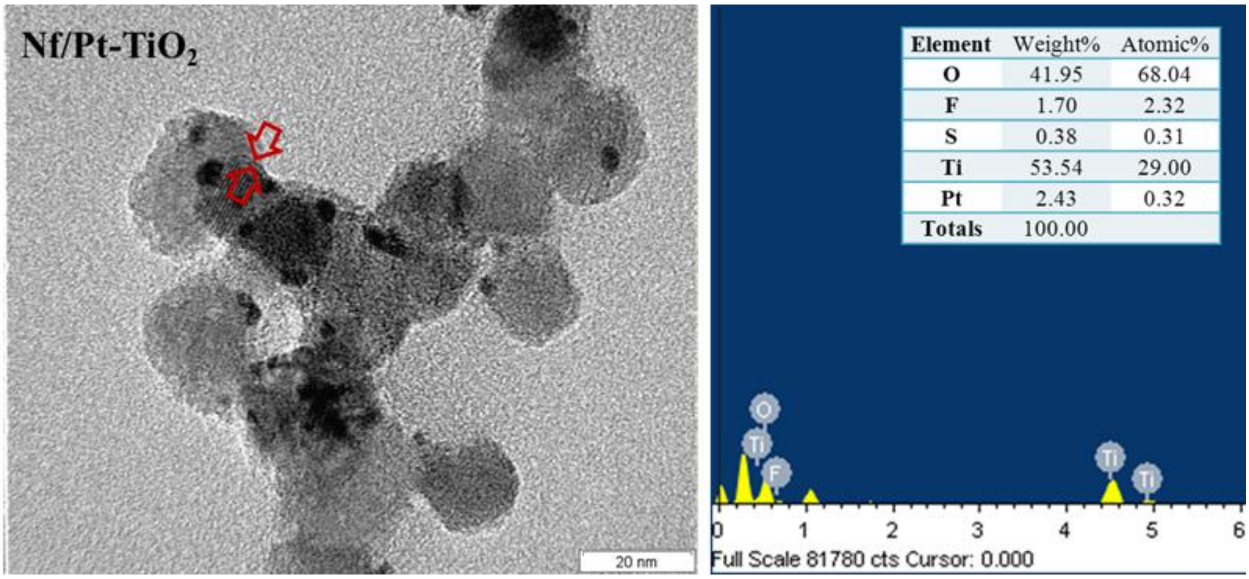

Figure S2. Micrographs for Nafion-coated Pt-TiO$_2$ (**2**) showing (a) TEM image, and (b) EDS spectrum.

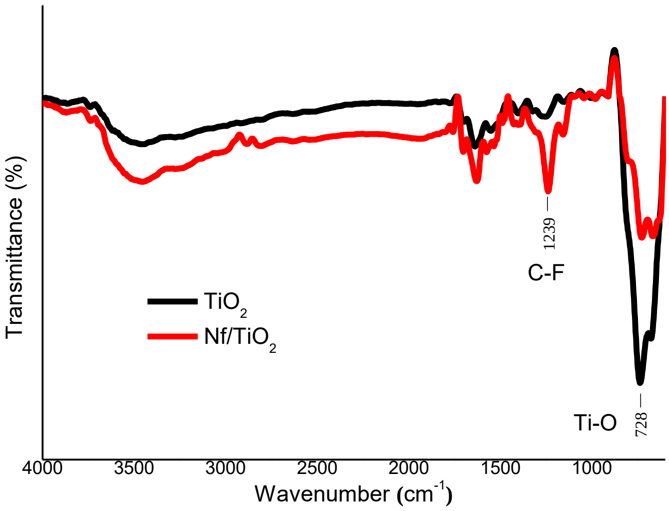

Figure S3. FT-IR spectra of platinized TiO$_2$ (**1**) and Nafion-coated Pt-TiO$_2$ (**2**).

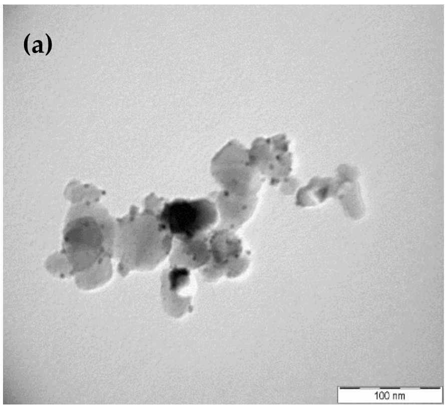

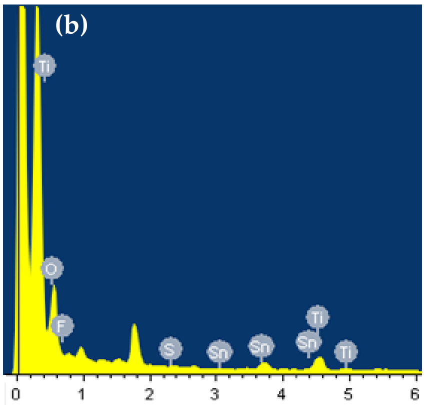

**Element**	Weight%	Atomic%
**O**	48.90	69.33
**F**	12.90	15.40
**S**	2.30	1.63
**Ti**	24.04	11.38
**Sn**	11.86	2.27
**Totals**	100.00	

Figure S4. Micrographs for SnP^{6+}-embedded Nafion/Pt-TiO_2 (**3**) showing (a) TEM image, and (b) EDS spectrum.
